# Factors explaining the yearly changes in minimum bottom dissolved oxygen concentrations in Lake Biwa, a warm monomictic lake

**DOI:** 10.1038/s41598-018-36533-7

**Published:** 2019-01-22

**Authors:** Takehiko Fukushima, Tomohiro Inomata, Eiji Komatsu, Bunkei Matsushita

**Affiliations:** 1 Faculty of Life and Environmental Sciences, University of Tsukuba, 1-1-1 Tennodai, Tsukuba, 305-8572 Japan; 2 Ibaraki Kasumigaura Environmental Science Center, 1853 Okijyuku-machi, Tsuchiura, 300-0023 Japan; 3 LERCS Co., 5-80 Aioi-cho, Naka-ku, Yokohama, 231-0012 Japan

## Abstract

Vertical profiles of dissolved oxygen (DO) and water temperature (WT) measured bi-monthly for 36 years (1980–2015) near the deepest part of a warm monomictic lake were analyzed with special reference to yearly minimum DO at bottom (DOmin). DOmin changed yearly (3.0 ± 1.2 mg l^−1^) and significant differences in DOmin were not observed between Period I (1980–1993; cooler and worse in water quality) and Period II (1994–2015; warmer and better in water quality). This unclear trend in DOmin was probably due to the offsetting influences between warming induced by global warming and oligotrophication attempted by local governments etc. for the study period. DOmin was positively correlated with disturbance time (timing of last cold water intrusion observed from Mar to Aug), which could be related to the start of DO depletion at bottom. Thus, the linear model using this parameter could predict yearly DOmin fairly well for the entire study period (r^2^ = 0.60). In addition, DOmin and time of disturbance were correlated negatively with water density at bottom in Jan and positively with water density equilibrated to air temperature (AT) in Mar. Higher lake water density after full depth mixing advances the disturbance time. In contrast, lower AT in Mar and/or higher density of influent water after Mar delays the time likely due to the larger amount of snowfall in the watershed. Further, DOmin was positively correlated with maximum wind velocity in Sep which probably induced the recovery of DO. Multiple-regression models to predict DOmin using these meteorological and water quality parameters were developed (r^2^ ≥ 0.38, worse performances than the model using disturbance time) to forecast future trends of DOmin through global warming and/or climate change. Significant influences of water or sediment oxygen demands on DOmin were not detected. We also discuss the applicability of the proposed models.

## Introduction

The dynamics of dissolved oxygen (DO) distribution in inland waters are fundamental to the development of an understanding of the distribution, behavior, and growth of aquatic organisms^1^. The DO state reflects the health of the lake environment. Recently, there have been concerns about the effects of global warming on the state of lake DOs, particularly bottom DO, through physical, chemical and biological processes^2^. As a result, the Japanese government has started to use bottom DO conditions as an environmental standard in lake and coastal water bodies^3^.

In European and North American lakes, particularly warm monomictic or dimictic lakes, depletion of DO in the hypolimnia during stratification has been observed and analyzed for more than 100 years^4,5^. The degree of such hypolimnetic DO depletion can be affected by the duration of the stratification period and the DO depletion rate^5^. In general, the DO deficit is expected to grow owing to eutrophication and warming^1^. Similarly, hypolimnetic waters have been reported to become anoxic in deep tropical lakes^6^. In response to these changes, a number of diverse approaches have been adopted to investigate the temporal dynamics and spatial patterns of hypoxia in European lakes and coastal regions^2^.

**Figure 1:**
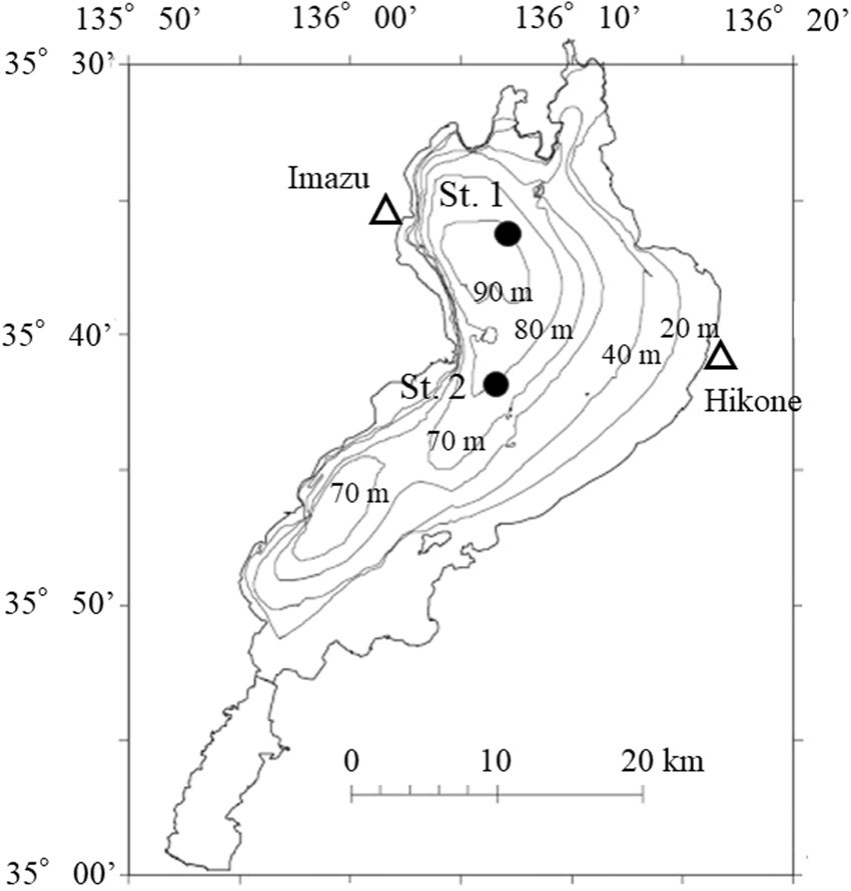
Lake Biwa, water-quality measuring stations (solid circles; St. 1: this study [LBERI]; St. 2: Sohrin *et al*.^8^ [Shiga Prefectural Fisheries Experiment Station]) and stations for measuring meteorological factors (open triangles; Imazu, Hikone). Reproduced with permission from Sohrin *et al*.^8^. This figure is not covered by the CC BY licence. [Credits to the Japanese Society of Limnology]. All rights reserved, used with permission.

In Lake Biwa (Fig. 1), the largest lake and most important freshwater resource in Japan, much scientific and social attention has been paid to bottom DO problems in recent decades. The north basin of Lake Biwa is itself a warm monomictic lake. Using limnological observation data up to 1971, Naka^7^ showed that the amount of DO in deep water before the season of full-depth mixing has been decreasing due to a progressing eutrophication. Sohrin *et al*.^8^ analyzed long-term changes in bottom water, and showed that, since 1999, yearly minimum DO concentrations <50 μmol kg^−1^ have started to occur frequently at around a depth of 90 m. Although they considered that these changes were most likely the result of global warming (S-Fig. 1) and local eutrophication, they did not specifically analyze the cause(s).

Fushimi^9^ suggested that a trend of decreasing snowfall in the catchment might be contributing to the decrease of DO in the deep layer. Based on an analysis of the vertical profiles of DO and influential factors, Tsujimura *et al*.^10^ and Jiao *et al*.^11^ reported that the DO conditions in the hypolimnion during the stratification period were affected by the occurrence of violent lake-water mixing (possibly due to meteorological events, e.g., typhoons), the species and amount of phytoplankton in the epilimnion in summer, and water temperature and DO concentration in spring. They also investigated the impact of a low DO condition on benthic organisms. Okamoto^12^ described the changes in bottom DO concentrations near the deepest part of the lake during specific years in great detail, and discussed the influencing factors. These analyses all considered that the yearly minimum DO might be related to the beginning of stratification, time of overturn, and rate of decrease of DO (DO decrease rate) under the stratification conditions. However, no report has quantitatively explained and/or predicted the yearly minimum DO in bottom water in recent decades.

The purpose of the present study was therefore to elucidate the factors affecting yearly minimum DO concentrations in bottom water using bi-monthly data from the last 36 years on the vertical distribution of water temperature and DO at the center of Lake Biwa. Much attention was paid to meteorological factors, since it is widely anticipated that such factors will reveal the influences of global warming and/or climate variation on lake DO conditions. Such information would be useful not only for building a detailed simulation model describing the water cycle, and physical, chemical and biological processes in the watershed and lake, but also when considering countermeasures for low DO problems.

## Results

### Seasonal and yearly change in DO and WT

**Figure 2:**
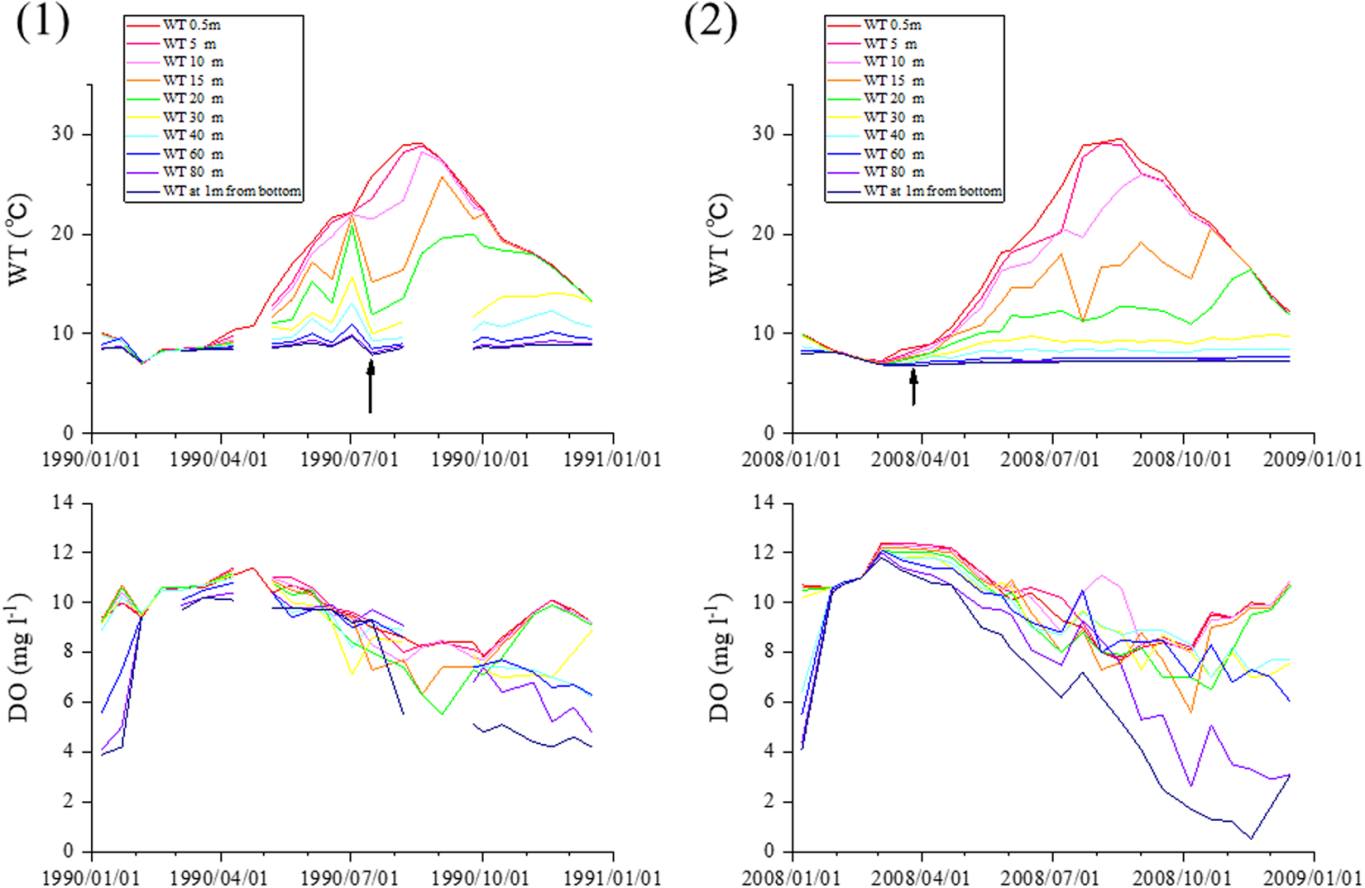
Examples of temporal changes in the vertical profiles of water temperature (WT) and dissolved oxygen (DO) in (1) 1990 and (2) 2008. An arrow indicates the time of the disturbance event (see text).

**Figure 3:**
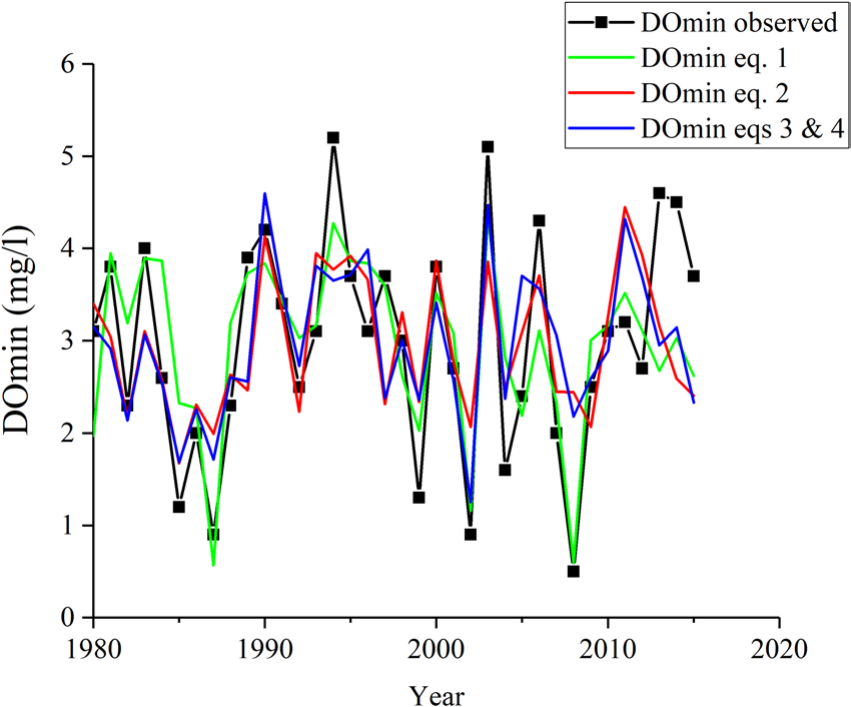
Yearly changes in minimum DO concentrations at bottom (DOmin: black line) and their predicted values using eq. (1) (green line; r^2^ = 0.59, adjusted r^2^ = 0.58, RMSE = 0.74 mg l^−1^), eq. (2) (red line; r^2^ = 0.38, adjusted r^2^ = 0.30, RMSE = 0.92 mg l^−1^) and eqs (3) and (4) (blue line; r^2^ = 0.44, adjusted r^2^ = 0.30, RMSE = 0.87 mg l^−1^).

The observed changes in DO and water temperature (WT) at the lake bottom are shown in S-Fig. 2. Seasonal cycles in DO are clearly indicated, and gradually increasing oscillations of several-year periods overlapping on seasonal cycles were observed in WT. Similar oscillations in WT were also observed at St. 2^8^. Examples of seasonal changes in vertical profiles of DO and WT for specific years are shown in Fig. 2 and S-Fig. 3. Generally, DO at bottom decreased monotonously from the start to the end of the DO depletion period, but occasional small disturbances (recoveries of DO) were also observed, e.g., Jul 2008 in Fig. 2 (2). This disturbance was possibly caused by a horizontal movement of bottom waters, but the details are unknown.

**Figure 4:**
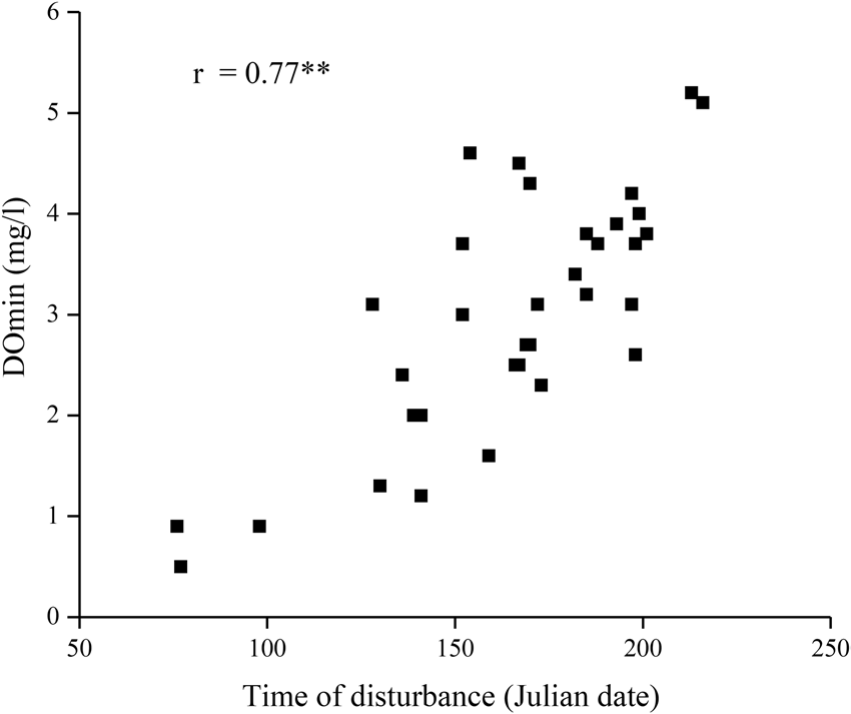
Relation between time of disturbance and DOmin.

As can be seen in Fig. 2 and S-Fig. 3, DO at bottom decreased substantially in 1987, 2002 and 2008, while the decreases in 1983, 1990 and 2014 were smaller. The starting times of DO decrease occurred earlier in the former years compared to the latter. WT changes appeared roughly similar in all six of these years, but the minimum WT at bottom (indicated by arrows in the figures) was observed sooner in the former years than in the latter. This timing of minimum WT at bottom seemed to influence the DO decrease at bottom. Thus, we call this the disturbance time, and analyze it below. Changes in the yearly minimum DO at bottom (3.0 ± 1.2 mg l^−1^: Fig. 3), the DO minimum date (S-Fig. 4), the WT of the disturbed water, and the date of disturbance did not show clear trends or cyclic oscillations (Table 1).

**Table 1:** Means and standard deviations of related water quality and meteorological parameters in the two study periods and significance of differences.

	I: 1980–1993	II: 1994–2015	
BOD (mg l^−1^)*1	0.72 ± 0.08	0.54 ± 0.08	P < 0.001
Chlorophyll *a* (μg l^−1^)*1	4.46 ± 0.92	3.50 ± 0.63	P < 0.001
TP (μg l^−1^)*1	9.4 ± 0.7	8.1 ± 0.7	P < 0.001
TN (mg l^−1^)*1	0.284 ± 0.025	0.280 ± 0.034	
NO_3_-N (mg l^−1^)*1	0.102 ± 0.016	0.112 ± 0.024	
DOmin (mg l^−1^)*2	2.81 ± 1.03	3.07 ± 1.29	
Date of DOmin (Julian date)*2	310 ± 31	316 ± 38	
Bottom WT at DOmin (°C)*2	7.3 ± 0.9	7.5 ± 0.4	
Av. of Surface WT from Aug to Oct (°C)*2	24.3 ± 0.9	25.3 ± 0.8	P < 0.01
Av. of bottom WT from Aug to Sep (°C)*2	7.1 ± 0.9	7.3 ± 0.4	P < 0.001
Yearly minimum bottom WT (°C)*2	6.2 ± 1.0	6.9 ± 0.4	P < 0.01
WT of disturbance water (°C)*2	6.5 ± 1.1	7.3 ± 0.4	P < 0.001
DO of disturbance water (mg l^-1^)*2	9.1 ± 0.7	8.9 ± 0.8	
Date of disturbance (Julian date)*2	167 ± 35	163 ± 34	
DO decrease rate from Apr to Sep (mg l^−1^ d^−1^)*2	0.034 ± 0.009	0.037 ± 0.007	
AT in Mar (°C)*3	5.9 ± 1.3	6.2 ± 1.0	
Av. of AT from Mar to Oct (°C)*3	17.5 ± 0.6	18.4 ± 0.5	P < 0.001
Num. of cold days from Oct 1 to Nov 15 (see text)*3	14.0 ± 3.5	8.3 ± 4.2	P < 0.001
SA (mm) from Nov to Apr*3	296 ± 161	277 ± 153	
Max WV (m s^−1^)*4	9.4 ± 3.2	9.8 ± 2.8	

*1Annual (from Apr to Mar) average for the surface water samples (0.5 m) at 28 stations in the North Basin of Lake Biwa, *2: St. 1, *3: Imazu, *4: Hikone.

Abbreviations are explained in the text. P indicates statistical significance.

### Relations between the yearly minimum DO and influencing factors

Results of the correlation analysis between yearly minimum DO concentrations and yearly values of the factors possibly influencing it were as follows (*P < 0.05, **P < 0.01). At first, the yearly minimum DO (DOmin) was not significantly correlated with specific WT or DO values, e.g., minimum WT at bottom, WT at bottom when the minimum WT at the surface was observed, DO when the minimum WT at the surface was observed, and WT when the minimum DO was observed. DOmin showed non-significant correlations with the dates of the unusual WT or DO values, e.g., the dates when minimum WT at the surface, minimum WT at bottom, and DOmin were observed. In addition, non-significant correlations were found between DOmin and the periods indicating DO depletion duration, e.g., the periods from the time of minimum WT at the surface or bottom to the time when DOmin was detected (the time of DOmin).

With regard to meteorological factors in the spring, each of the following showed a higher correlation with DOmin in a spring month compared to other months: air temperature (AT) in Mar, precipitation (PR) in Apr, and sunshine hours (SH) in Apr at Imazu. However, none of these correlations were statistically significant. The snowfall amount (SA) at Imazu during winter showed a positive correlation with DOmin, but this relation was also not significant. As to the values in fall, the correlation coefficients with AT in Sep and monthly maximum wind velocity averaged for 10 minutes (max WV) at Hikone in Sep (r = 0.34*; r: correlation coefficient) were high. The number of cold days did not have a significant influence on DOmin. Regarding WT at bottom, this parameter was significantly correlated with DOmin at the Jan 1^st^ survey (r = 0.43**) and Jan 2^nd^ survey (r = 0.33*), but the correlations with WT in the fall, e.g., WT at the surface at the Oct 1^st^ survey were non-significant. In addition, a significant correlation was found between DOmin and the DO decrease rate from Apr 1^st^ to Sep 2^nd^ survey (r = −0.49**).

**Figure 5:**
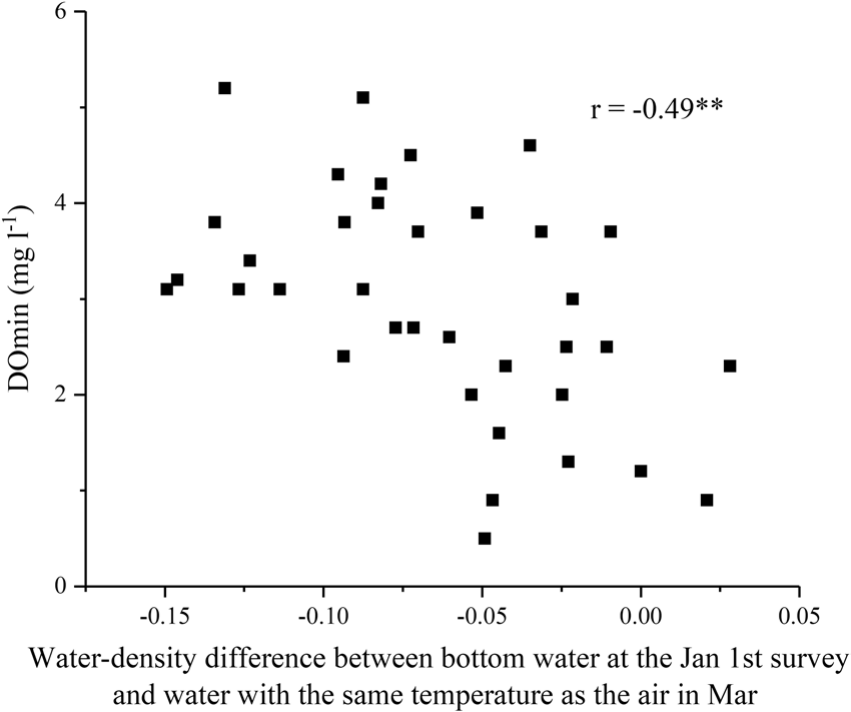
Water-density difference between bottom water at the Jan 1^st^ survey (see text) and water with the same temperature as the air in Mar vs. DOmin.

Because the density of water (ρ) (a function of water temperature as shown in the Methods) has a more direct influence on the mixing process in lakes than temperature, we analyzed the correlation between DOmin and water density. Compared to temperature, higher correlations were found between DOmin and ρ as a function of WT at the Jan 1^st^ survey (r = −0.43**), and DOmin and ρ of water equilibrated to AT in Mar. As suggested in the previous section, the disturbance time could affect DOmin. In fact, the relationship between disturbance time and DOmin showed the highest correlation in this study (Fig. 4; r = 0.77**). Because both low water density in winter (Jan 1^st^ survey) and high water density in early spring (Mar) (r = −0.34*) (i.e., low AT in Mar [r = 0.36*]) delay the disturbance time, the difference between them might be a good estimator of the disturbance time (S-Fig. 5; r = −0.40*) and thereby the DOmin (Fig. 5; r = −0.49**).

The correlation between DOmin and yearly averaged water quality—e.g., biochemical oxygen demand (BOD), chlorophyll *a*, total phosphorus (TP)—was not significant. The rate of decrease in DO from the Apr 1^st^ to Sep 2^nd^ survey was significantly correlated with the rates of decrease for other long periods (i.e., periods of more than 3 months), but not with the rates of decrease for short periods (less than 2 months). The DO decrease rate from the Apr 1^st^ to Sep 2^nd^ survey showed positive correlations with ATs, but those correlations were not significant.

### Water quality and air temperature trends during the survey period

**Figure 6:**
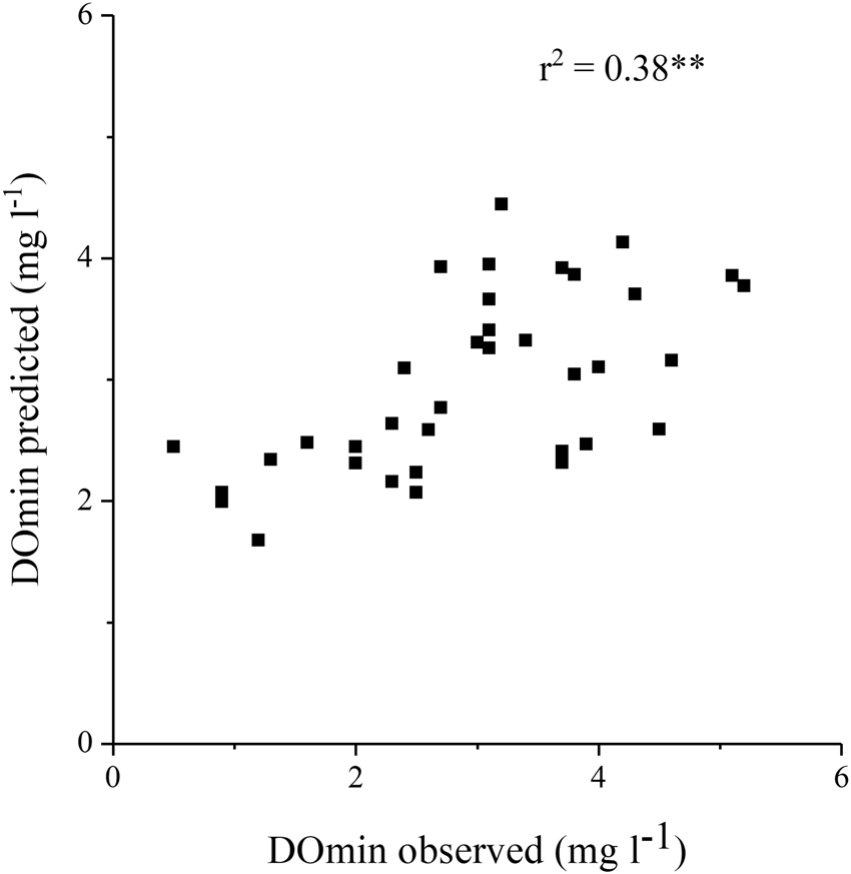
Relation between observed DOmin and predicted DOmin using eq. (2).

In the north basin of Lake Biwa, an oligotrophication trend was observed for the period 1980–2015, as shown in S-Fig. 6 ^13^. In addition, the shift analysis indicated clear changes between 1993 and 1994 for BOD and TP and between 1999 and 2000 for chlorophyll *a*. Thus, we divided the whole period into two parts: Period I from 1980–1993 and Period II from 1994–2015. There were significant differences between the two periods in the concentrations of BOD, chlorophyll *a*, and TP, averaged surface WT from Aug–Oct., yearly minimum bottom WT, average of bottom WT from Aug–Sep, WT of the disturbed water, average of AT from Mar to Oct, and number of cold days from Oct 1 to Nov 15 (Table 1). In Period II, the concentrations of all the parameters related to water quality (BOD, chlorophyll *a*, TP) were lower except the nitrogen level. WTs except WT at DOmin and ATs were higher in this period. In general, lake water quality was better and the lake environment was warmer in Period II than in Period I.

Annual averaged air temperature increased at a rate of 0.039 °C y^−1^ (the regression coefficient in the linear model with year) during the period from 1980–2015. The increase rates were different month-to-month (S-Fig. 2 (2)). Significantly larger increases were observed from May–Oct, while smaller, non-significant increases were observed in other months.

### Prediction of DO minimum in the bottom layer

First, models were constructed using the variable (i.e., the disturbance time) that had the highest correlation coefficient with DOmin in this study. Its linear, quadratic and cubic equations were compared based on the adjusted r^2^ values and then the following linear one was selected (n = 36, r^2^ = 0.60**, adjusted r^2^ = 0.58, RMSE = 0.74 mg l^−1^):

1 $${\rm{DOmin}}=0.027\times {\rm{Tdisturbance}}-1.484$$

where Tdisturbance is disturbance time (Julian date). The characteristics of yearly changes were well described, but the amplitudes of the changes were somewhat smaller than the observed values due to the theoretical characteristics of regression model (Fig. 3).

Next, the multiple regression models to predict DOmin were examined using meteorological and water quality parameters. Based on the correlation analysis shown above, we compared the models with combinations of the following parameters as independent variables: WTs equilibrated with AT at Imazu in Feb and Mar, water densities of these WTs, bottom WTs during the first and second periods of Jan, bottom WTs during the first and second periods of Feb, water densities of these WTs, total sunshine hours at Imazu in Mar and Apr, precipitation at Imazu in Apr, monthly max WV averaged for 10 minutes in Sep at Hikone, and ATs at Imazu in Jul, Aug, Sep and Oct. Then, the following model was chosen to give the highest adjusted r^2^ for all data (1980–2015) (n = 36, r^2^ = 0.38**, adjusted r^2^ = 0.30, RMSE = 0.92 mg l^−1^: Figs 3 and 6):

2 $$\begin{array}{rcl}{\rm{DOmin}} & = & -6.826\times {\rm{\rho }}^{\prime} ({\rm{WTJ1}})+16.733\times {\rm{\rho }}^{\prime} ({\rm{WT}}({\rm{AT3}}))\\  &  & -0.011\times \,{\rm{SH}}4+0.124\times {\rm{W}}9+3.54\end{array}$$

where DOmin is mg l^−1^, ρ′: (ρ − 1) × 10^3^, ρ is the water density corresponding to the water temperature (g cm^−3^), WTJ1 is the bottom WT during the first period of Jan, WT(AT3) is the WT equilibrated with AT at Imazu in Mar, SH4 is the total sunshine hours at Imazu in Apr (hr), and W9 is the monthly max WV averaged for 10 minutes in Sep at Hikone (m s^−1^).

Similar to eq. (1), this model gave somewhat smaller variations compared with the observed ones due to the rather small regression coefficient, and the discrepancies were larger in the latter period compared with the former. To clarify the differences between the periods, we applied multiple regression analysis separately for Periods I and II. The models obtained for the highest adjusted r^2^ were eq. (3) (1980–1993) (n = 14, r^2^ = 0.61**, adjusted r^2^ = 0.44, RMSE = 0.61 mg l^−1^) and eq. (4) (1994–2015) (n = 22, r^2^ = 0.37**, adjusted r^2^ = 0.22, RMSE = 1.01 mg l^−1^) for Periods I and II, respectively.

3 $$\begin{array}{rcl}{\rm{DOmin}} & = & -6.109\times {\rm{\rho }}^{\prime} ({\rm{WTJ1}})+9.760\times {\rm{\rho }}^{\prime} ({\rm{WT}}({\rm{AT3}}))\\  &  & -0.015\times \,{\rm{SH4}}+0.109\times {\rm{W}}9+4.14\end{array}$$

4 $$\begin{array}{rcl}{\rm{DOmin}} & = & 12.757\times {\rm{\rho }}^{\prime} ({\rm{WTJ1}})+16.993\times {\rm{\rho }}^{\prime} ({\rm{WT}}({\rm{AT}}3))\\  &  & -0.387\times \,{\rm{AT7}}+0.120\times {\rm{W}}9+10.67\end{array}$$

In eq. (3), the same parameters were chosen as in eq. (2). In eq. (4), a new parameter AT7 (air temperature in Jul) (°C) was used instead of SH4. The correlation between the predicted and observed DOmin (S-Fig. 9; combined eqs (3) and (4); r^2^ = 0.44**, adjusted r^2^ = 0.30, RMSE = 0.87 mg l^−1^) was better than the correlation shown in Fig. 6, and higher amplitudes of change were obtained. Particularly in Period I, better agreement was achieved.

## Discussion

### Characteristics of long-term trends in the DO minimum in bottom water

Sohrin *et al*.^8^ reported that the bottom DO at St. 2 has been decreasing since 1959. They considered that the increases in bottom WT and nutrient concentrations (NO_3_-N, PO_4_-P, and Si(OH)_4_) were responsible for the decrease in DO. The relationships between yearly minimum DO concentrations at St. 1 and St. 2 appeared to be rather scattered, but were significantly correlated (S-Fig. 7).

In this study, we could not identify a clear trend in the bottom DO concentration (Fig. 3; Table 1). The difference in the periods investigated likely affected the difference in bottom DO trends and main factors influencing the DOmin. We therefore statistically analyzed the data from Sohrin *et al*.^8^ by separating the whole period from 1963 to 2012 into three periods—(A) 1963–1979, (B) 1980–1993, and (C) 1994–2012—and compared the data from Periods (A), (B) and (C) with those from our present Periods I & II. In Period (A), organic pollution and eutrophication progressed year by year through postwar economic development. The local government (Shiga Prefecture) enacted the Ordinance for Prevention of Eutrophication of Lake Biwa with the enthusiastic cooperation of the residents in 1979, e.g., construction of sewerage facilities and enactment of nutrient regulations of factories, commercial facilities, and household detergents^14^. Due to such attempts, many parameters of water quality began to improve after the late 1990s^13,14^. There were gradual increases in WT and NO_3_-N concentrations after the late 1990s, but the changes in bottom DO after 1980 were not significant (S-Table 1). In addition, there were significant correlations between DOmin and minimum bottom WT (r = −0.31*), and between DOmin and yearly averaged NO_3_-N (r = −0.55**) for data between 1963 to 2012, but the correlations between DOmin and minimum bottom WT and between DOmin and yearly averaged NO_3_-N were not significant for the period between 1980 and 2012.

In short, the study period (1980–2015) exhibited overall trends of gradual oligotrophication and warming, as discussed in the previous section and shown in Table 1. The unclear trend in yearly DOmin is probably attributable to the offset between suppression of DO deficit by oligotrophication and its intensification by warming during our study period.

### Mechanism underlying the DO minimum in bottom water

Theoretically, DOmin concentrations at the bottom of warm monomictic and dimictic lakes are determined by the duration of the stratified period, i.e., from its start to its end, and the DO depletion rate (e.g., Walker^5^). However, significant correlations between DOmin and DO depletion periods were not obtained in this study. The rates of decrease of DO over long periods (more than three months) were significantly negatively correlated with DOmin. However, this correlation was merely due to different characteristics of the same phenomena, because these DO decrease rates were calculated as the amount of decrease in DO (closely related to DOmin in the case of long period) divided by the duration of the period.

In contrast, the decrease rates for shorter periods (less than two months) did not show a clear influence on DOmin, indicating the scattering of the DO decrease rates during the stratification period. Tsujimura *et al*.^10^ reported a significant correlation between the average volumes of phytoplankton cells from Mar to Sep and DOmin (r = −0.57*). In this study, we did not include such a parameter explicitly due to the imperfect availability of data over the whole study period. In eq. (4), AT in Jul was selected as one of the independent variables for the prediction of DOmin in Period II, suggesting that this parameter is related to the DO decrease rate.

Further, the sediment oxygen demand (SOD) might contribute to the determination of the value of DOmin^4^. There has been no report on SOD in the north basin of Lake Biwa. Gelda *et al*.^15^ built a sediment diagenesis model expressing carbon and oxygen dynamics in water and sediment and then predicted the change in SOD over a 30-year period in Onondaga Lake. However, yearly estimation of SOD is quite difficult without deliberate observation or a dynamic model. Rucinski *et al*.^16^ expressed SOD as a function of TP and then predicted the hypoxia in Lake Erie. Adopting their approach, we added TP to eqs (1)–(4) as an independent variable, but this yield no improvement in the adjusted r^2^. In addition, regression equations similar to eq. (1) were obtained separately for Periods I and II. There was no significant difference between these equations, indicating that the difference in SOD between the periods was not explicitly expressed in these equations. This may have been because there was an insignificant difference in SOD between the two periods, or because SOD did not have a significant influence on DO change during the isolation period of bottom water after the last intrusion of cold water. In any case, in order to accurately forecast the future hypolimnetic DO conditions in this lake, it would be necessary to track and predict the long-term change in oxygen consumption rates in water and sediment.

In Lake Biwa, the behavior of the hypolimnetic DO has been discussed from the perspective of the timing of complete oxygenation of the bottom water, the WT at that time, SA in the basin, events such as strong winds (e.g., typhoon), cold days in autumn, and phytoplankton species and their amounts in summer^9–12,17^. In this study, we analyzed the relations between DOmin and these influencing factors, and found that a limited number of factors (WT at the Jan 1^st^ survey, WT at the Jan 2^nd^ survey, max WV in Sep, water density as a function of AT in Mar), were significantly influential, but not predominant expressed by the relatively low regression coefficients in eqs (2)–(4).

However, the quite high correlation (Fig. 4: r = 0.77**) with the disturbance time, which determines the start of DO depletion at bottom, seemed likely to have affected the DOmin at bottom. In this sense, the timing of the last intrusion of cold water, when DO is plentiful (DO of the disturbed water: 9.0 ± 0.8 mg l^−1^), would be the start of DO depletion at bottom. The rather small variations in DO of the disturbed water indicated that the DO state was relatively constant at the start. Yoshiyama *et al*.^17^ suggested the importance of gravity currents, as well as the contribution of convective mixing to the delivery of oxygen to the profundal zone of the north basin of Lake Biwa. Because a high correlation was observed between AT in Mar and SA at Imazu (r = −0.61**), low AT in Mar (i.e., high water density equilibrated to AT in Mar) could delay the disturbance time by melting the snow in the watershed more slowly and increasing the density of the influent water after Mar. It would be reasonable to consider that both the water density state in lakes after full-depth mixing and the water density of influent water after Mar affects the disturbance time, and therefore, the density difference between them could be a good predictor of disturbance time (S-Fig. 5 and Fig. 5) and DOmin (r = 0.49**). This density difference was a better predictor of the DOmin than the difference of AT (r = 0.39*). Importantly, higher lake water density after full-depth mixing advances this disturbance time, and vice versa. In contrast, higher density of influent water after Mar due to lower AT in Mar delays the time and vice versa.

Surprisingly, there was a positive, albeit non-significant, correlation between DOmin and its timing (i.e., the end of DO depletion). This result appears to indicate that, with respect to DOmin, the starting time of DO depletion is more important than the time of ending time. Because dissolved and particulate organic matter decompose logarithmically in lakes^18,19^, the initial processes (e.g., supply of organic matter to the bottom water) were suggested to have a great influence on the decrease compared with the subsequent processes. Only max WV at Hikone in Sep significantly raised the DOmin. Jiao *et al*.^11^ showed that the wind impact index (defined as the square of wind velocity divided by Schmidt’s Stability Index) affected the recovery of DO depletion. Okamoto^12^ found that a typhoon passing near the lake accompanied by strong winds in autumn affected DO conditions at the bottom. In addition, wind velocity at Imazu had a positive, although non-significant, correlation with DOmin, suggesting that the wind in flat areas more strongly affects the mixing in the lake compared with the winds in mountainous areas.

Meanwhile, there was a close relationship between AT for Sep to Dec in the preceding year and WT in the Jan 1^st^ survey (S-Fig. 8). A seasonally nonuniform increase in AT during our investigated period (e.g., a higher increase from Sep to Dec compared with Mar) could have resulted in a negligible delay in the start time of DO depletion in spite of the significant rise of annual mean AT. Thus, the seasonality of AT increase could affect the change in DOmin. For example, a change in WTJ1 from 7.7 °C (average for 1980–2015) to 8.7 °C would bring about an increase of 0.45 mg l^−1^ DOmin using eq. (2), assuming that other meteorological conditions were not varied. Future studies considering such phenomena will be necessary to understand and predict the influence of global warming and/or climate change on lake environments.

### Differences in the model predictability between Period I and II

The model agreement in Period I was better than that in Period II. Similar to eq. (1), linear models predicting DOmin were determined separately for Periods I and II. The RMSE (0.62 mg l^−1^) of the obtained model for Period I (Y = 0.0225 × −0.949 where X is the disturbance time and Y is the DOmin) was smaller than that (0.76 mg l^−1^) for Period II (Y = 0.0306 × −1.929), indicating a closer relationship with the disturbance time in Period I compared with Period II.

In the south basin of Lake Biwa, submerged plants hardly existed from 1960 to 1993, but they began to recover after the serious shortage of water in the summer of 1994^20^. Along with this recovery, there was an improvement of the transparency. It would be possible, albeit unlikely, that this regime shift occurring in the south basin led directly to the shift of water quality observed in the north basin, because there were large differences in lake morphology and water quality between the basins.

Kishimoto *et al*.^21^ reported that the average cell size of the phytoplankton community in the north basin decreased from 269 μm^3^ cell^−1^ in the 1980s to 56 μm^3^ cell^−1^ in the 2000s; in particular, the proportions of small nanoplankton (mainly Cyanophyceae) increased in summer. After the year 2000, the yearly variations in the summer phytoplankton composition increased. Yamada *et al*.^22^ indicated that the biodegradability of dissolved organic matter released from phytoplankton differed species by species based on experiments using *Microcystis aeruginosa*, *Staurastrum dorsidentiferum*, and *Cryptomonas ovata* under the water conditions existing in the north basin. These facts indicate that the larger yearly variability in the phytoplankton community for Period II compared to Period I resulted in less agreement in the DOmin prediction models. Therefore, biological information is necessary to improve the models, particularly in Period II.

### Applicability of the DOmin-predicting model and future study

The prediction model in eq. (1) has a fairly simple form and shows a good performance; thus a bi-monthly monitoring of bottom WT and DO can provide an early warning signal of low DOmin several months before the event. However, this model cannot forecast the future trends of DOmin through global warming and/or climate change. In contrast, the prediction models in eqs (2)–(4) are less accurate, but they can roughly forecast the future trends in DOmin corresponding to climate change. These models could be used to evaluate more elaborate simulation models describing the water cycle and the physical, chemical and biological processes in watersheds and lakes. However, we should keep in mind that eqs (3) and (4) are essentially disadvantageous for the prediction of future lake conditions.

At any rate, additional efforts will be needed to enhance the accuracy of these prediction models. Such efforts would be expected to include the collection of DO measurements with higher temporal and spatial resolution, meteorological data at more sites, and information on DO demand by water and sediments.

## Methods

### Lake Biwa

Lake Biwa, located at the center of Honshu Island, is the largest lake (area 670 km^2^; maximum depth, 104 m) in Japan (Fig. 1). One of several ancient lakes^23^, it contains more than 50 endemic species (benthic fauna and deep-living fish), any of which may be vulnerable to low oxygen concentrations in profundal habitats^17^. Lake Biwa has two basins—one to the south and one to the north. The south basin is shallow, with a mean depth of 4.0 m, while the north basin is deep, with a mean depth of 43 m. Judging from the trophic boundaries proposed by Carlson and Simpson^24^, the current trophic level of the north basin is between oligotrophic and mesotrophic in terms of total phosphorus, and mesotrophic in terms of chlorophyll *a*. This basin is warm monomictic; a clear thermocline is formed in summer and fall, and full depth mixings occur in late fall and spring.

### Survey and database

A bi-monthly limnological survey (1^st^ and 2^nd^ surveys in the respective months) has been conducted at the center of Imazu-oki (St. 1 in Fig. 1: 35°23′41″N, 136°07′57″E, ~90 m depth) from Apr 1979 to the present by LBERI (Lake Biwa Environmental Research Institute). WT and DO have been measured at 0.5, 5, 10, 15, 20, 30, 40, 60, 80 m below the surface and at 1 m from the bottom sediment (hereafter, at bottom) using a Hydrolab Quanta Multi-Probe Meter (Hydrolab, Loveland, CO) calibrated by JIS k02102-32. During the period from 1980–2001, WT and DO 1-m above the sediment were measured after the system was laid on the sediment and then raised 1 m. After 2002, a system with a depth sensor was used, and values 1-m above the bottom were determined after data processing.

The analysis in this study used the data from 1980–2015, thus, 36 years of data were analyzed. During the periods when the observations were more frequent than bi-monthly, we excluded any irregularly timed observations (there were only a few such occasions over the 36-year observation period). There were also a few periods when the observations were less than bi-monthly, and approximately 10 occasions when no measurements were conducted at several depths. During these periods, we did not interpolate the values except for special analyses (i.e., determination of the DO decrease rates, as shown below).

As for other water quality values—e.g., BOD, chlorophyll *a*, TP, total nitrogen (TN), and nitrate nitrogen (NO_3_-N)—the values averaged for the Japanese fiscal year (Apr to Mar) and in the north basin were used^13^. DO data at St. 2 (35°18′34″N, 136°07′19″E, ~80 m depth) in Fig. 1 measured by the Shiga Prefectural Fisheries Experiment Station were also used for comparison^8^.

Daily data on meteorological factors, including AT, PR, SH, SA, max WV at Imazu, and max WV at Hikone (Fig. 1) were used for the analysis^25^. The changes in monthly averaged AT at Imazu are shown in S-Fig. 1.

### Water density

Based on the database^26^, the following quadratic function was used to calculate the water density (y: g cm^−3^) from water temperature (x: 0 to 10 °C).

$${\rm{y}}=-\,0.0000077912{{\rm{x}}}^{2}+0.0000630498\,{\rm{x}}+0.9998445290$$

### Indices for characterizing WT and DO changes and meteorological conditions

The following indices for water quality were determined and used for further analysis: (1) the yearly minimum DO at bottom (sometimes including the data from Jan in the following year depending on DO seasonal change) and its date (all dates are Julian); (2) the DO decrease rate (several periods); (3) the WT and date (referred to as disturbance time) of the bottom water disturbance event (after Apr; WT at bottom layer was lower than before and after the event; more than 8 mg l^−1^, i.e., nearly DO-saturated water); (4) the yearly minimum WT at the surface and bottom waters and their dates. As a meteorological index, we calculated the number of days for several different periods during which the daily minimum AT was colder than the critical WT (number of cold days). The critical WT was that at the bottom when the yearly DO minimum was observed.

### Statistical methods

Statistical analyses were used to determine the correlation, the differences between the means (t-test) and the multiple regression for significance at the level of 0.05 (*) or 0.01 (**) (Excel-Statistics for 2016: BellCurve Social Survey Research Information Co., Tokyo). The values of adjusted r^2^ were used to select the independent parameters in multiple regression analysis. The root mean square errors (RMSE) were also calculated to evaluate the obtained prediction models.

$${{\rm{adjusted}}{\rm{r}}}^{{\rm{2}}}=1-(1-{{\rm{r}}}^{2})({\rm{N}}-1)/({\rm{N}}-{\rm{p}}-1)$$

$${\rm{RMSE}}=\sqrt{\frac{{\sum }_{{\rm{i}}=1}^{{\rm{N}}}{({{\rm{X}}}_{{\rm{pred}},{\rm{i}}}-{{\rm{X}}}_{{\rm{meas}},{\rm{i}}})}^{2}}{{\rm{N}}}}$$

Here, r^2^ is the square of the correlation coefficient (determination coefficient), N is the number of data, p is the number of independent variables, and ${{\rm{X}}}_{{\rm{pred}},{\rm{i}}}$ and ${{\rm{X}}}_{{\rm{meas}},{\rm{i}}}$ are the values of predicted and measured values, respectively.

The sequential t-test regime shift detection software ver. 3.2^27^ was used to determine shifts in the time series (significance level = 0.1; cut-off length = 10; Huber’s weight parameter = 1).

## Electronic supplementary material

Supplementary information
