# Supplementary material for: Factors explaining the yearly changes in minimum bottom dissolved oxygen concentrations in Lake Biwa, a warm monomictic lake
Source: Sci Rep. 2019 Jan 22;9:298. doi: 10.1038/s41598-018-36533-7 (PMC6342936; doi:10.1038/s41598-018-36533-7)
Supplement: Supplementary file 1 — Supplementary information [file 41598_2018_36533_MOESM1_ESM.pdf]

Factors explaining the yearly changes in minimum bottom dissolved oxygen concentrations in Lake Biwa, a warm monomictic lake

Takehiko Fukushima, Tomohiro Inomata, Eiji Komatsu, Bunkei Matsushita

S-Fig. 1 (1) Yearly changes in monthly averaged air temperature and (2) Air temperature increases in the respective months.

(1)

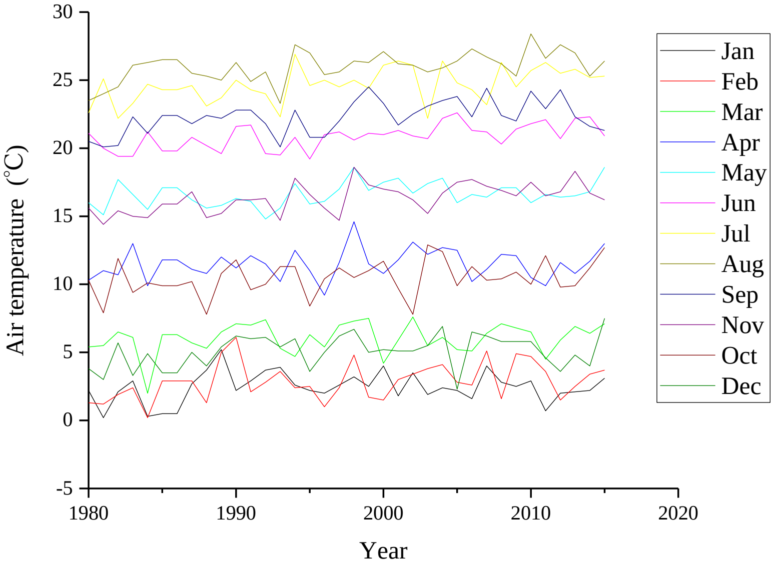

(2)

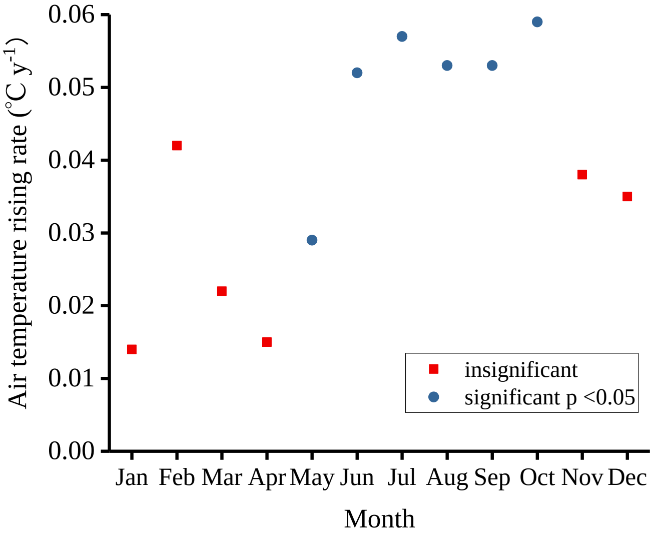

S-Fig. 2 (1) Temporal change in DO concentration at bottom and (2) temporal change in WT at bottom.

(1)

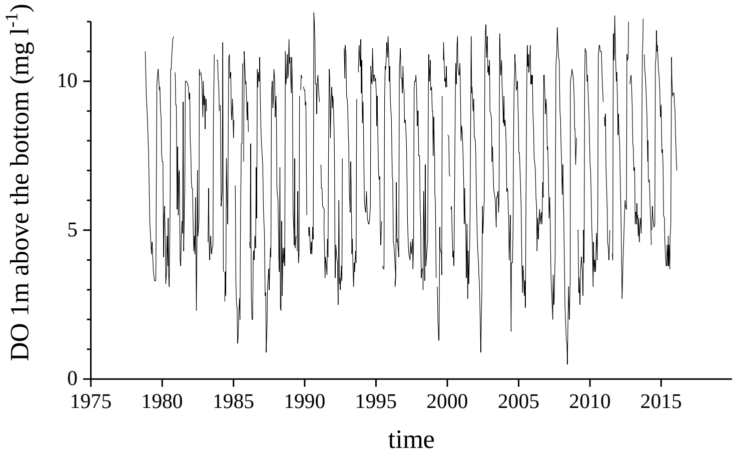

(2)

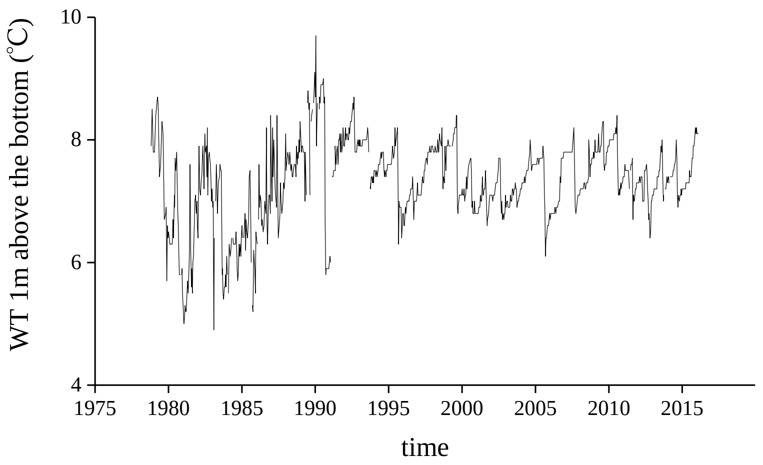

S-Fig. 3 Examples of temporal changes in vertical profiles of water temperature (WT) and dissolved oxygen (DO): (1) 1983; (2) 1987; (3) 2002; (4) 2014. An arrow indicates the time of disturbance event (see text).

(1)

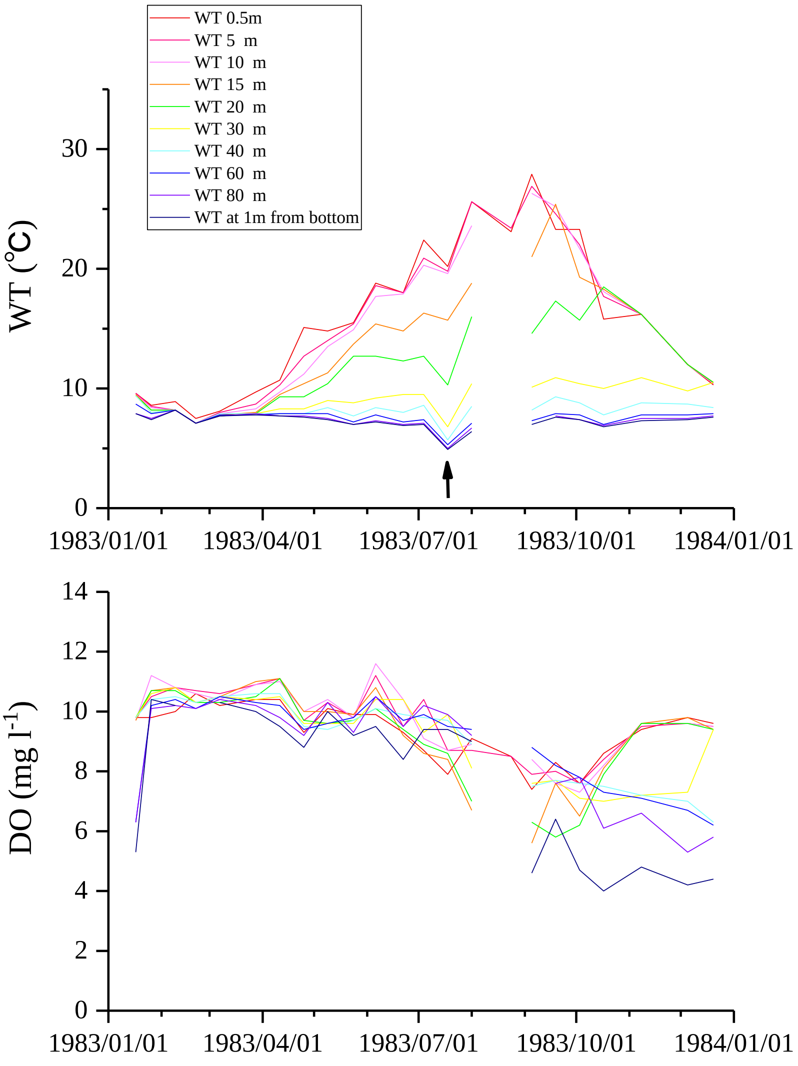

(2)

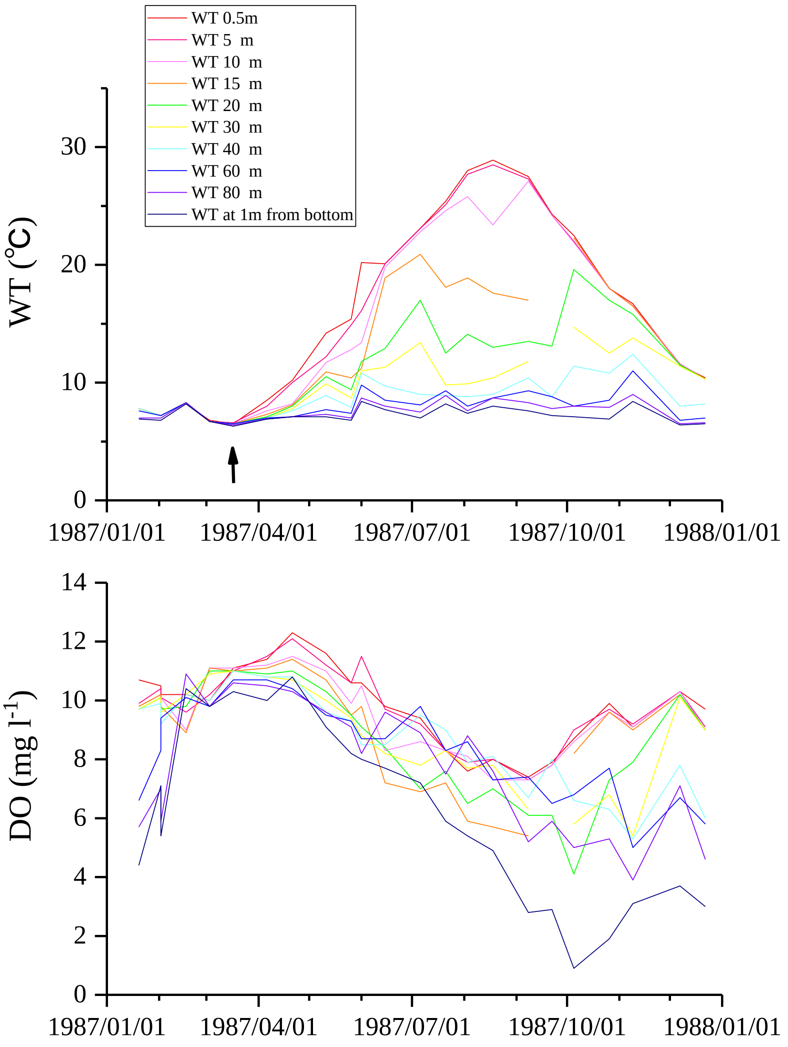
WT 0.5m
WT 5 m
WT 10 m
WT 15 m
WT 20 m
WT 30 m
WT 40 m
WT 60 m
WT 80 m
WT at 1m from bottom
WT (°C)
0
10
20
30
1987/01/01
1987/04/01
1987/07/01
1987/10/01
1988/01/01
DO (mg l-1)
0
2
4
6
8
10
12
14

(3)

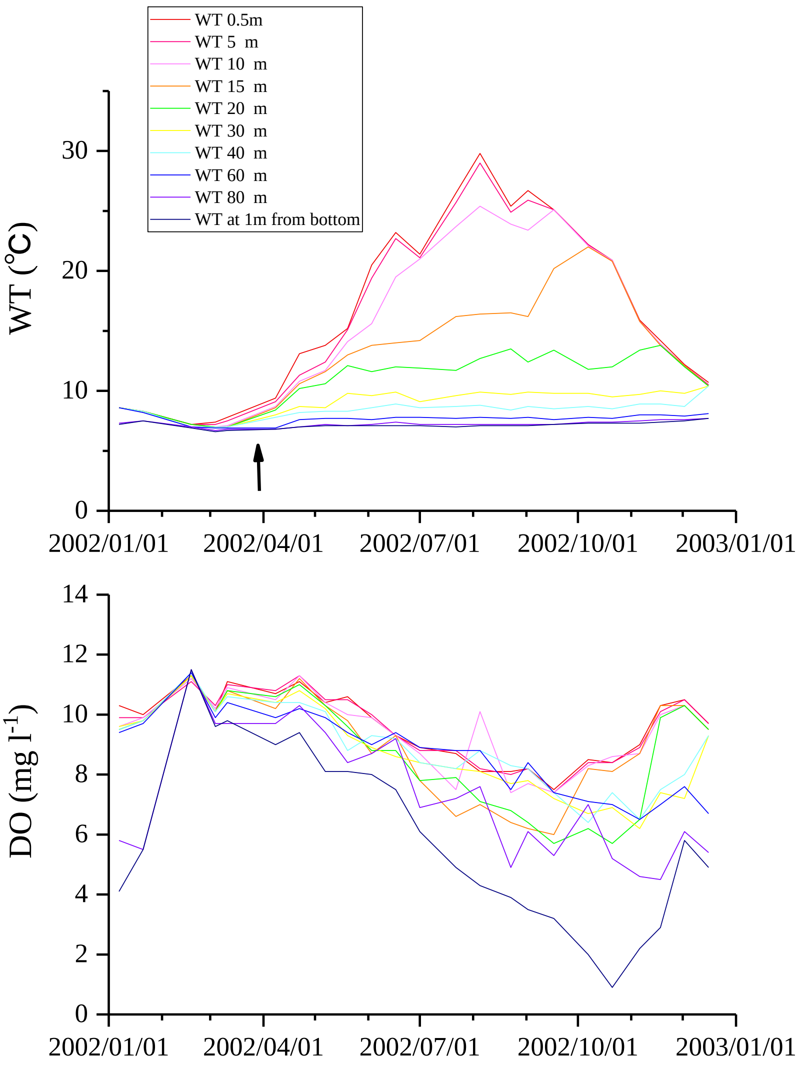
WT 0.5m
WT 5 m
WT 10 m
WT 15 m
WT 20 m
WT 30 m
WT 40 m
WT 60 m
WT 80 m
WT at 1m from bottom
WT (°C)
0
10
20
30
2002/01/01
2002/04/01
2002/07/01
2002/10/01
2003/01/01
DO (mg l-1)
0
2
4
6
8
10
12
14
2002/01/01
2002/04/01
2002/07/01
2002/10/01
2003/01/01

(4)

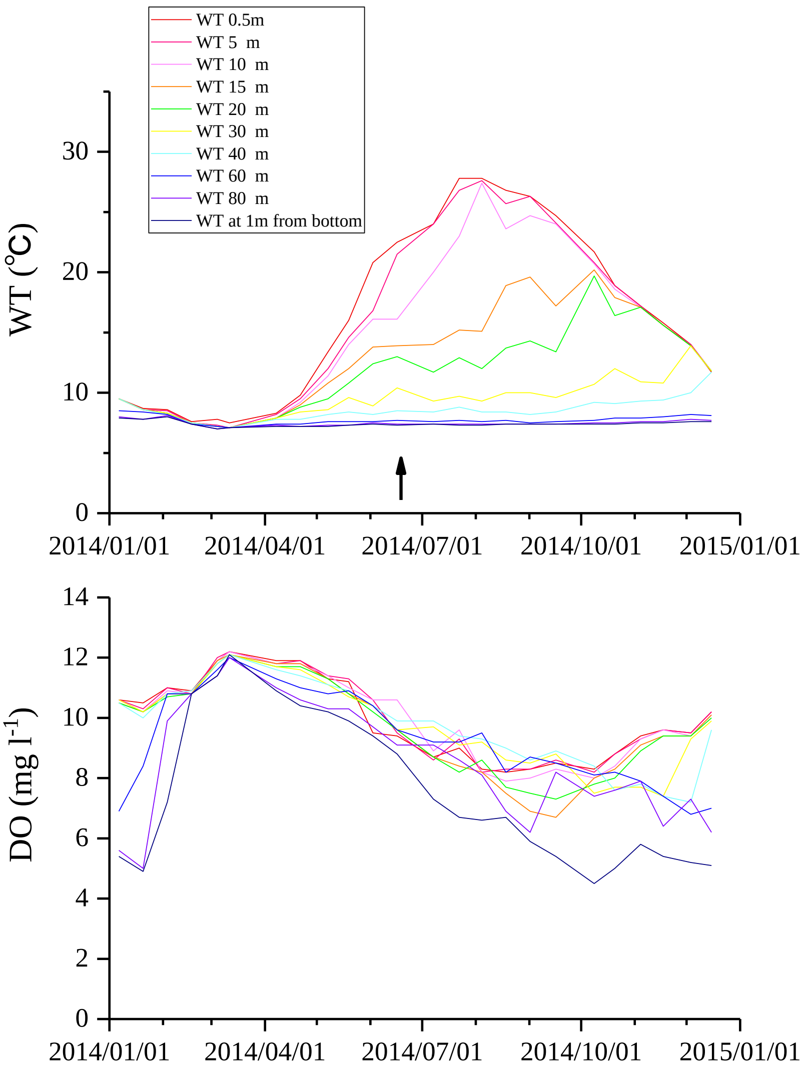

WT 0.5m
WT 5 m
WT 10 m
WT 15 m
WT 20 m
WT 30 m
WT 40 m
WT 60 m
WT 80 m
WT at 1m from bottom
WT (°C)
0
10
20
30
2014/01/01
2014/04/01
2014/07/01
2014/10/01
2015/01/01
DO (mg l-1)
0
2
4
6
8
10
12
14
2014/01/01
2014/04/01
2014/07/01
2014/10/01
2015/01/01

S-Fig. 4 (1) Temporal change in date of DOmin observed; (2) temporal change in DO decrease rate from Apr to Sep.

(1)

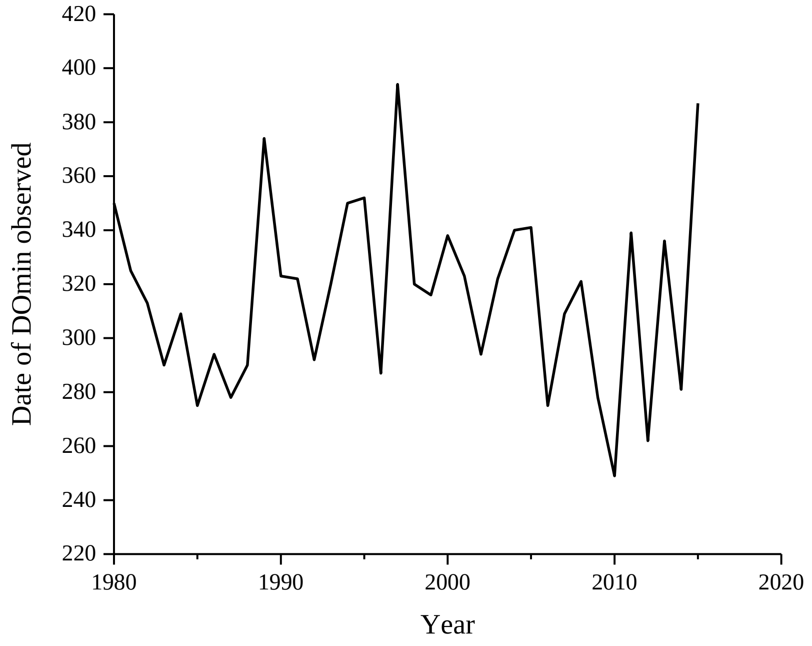

(2)

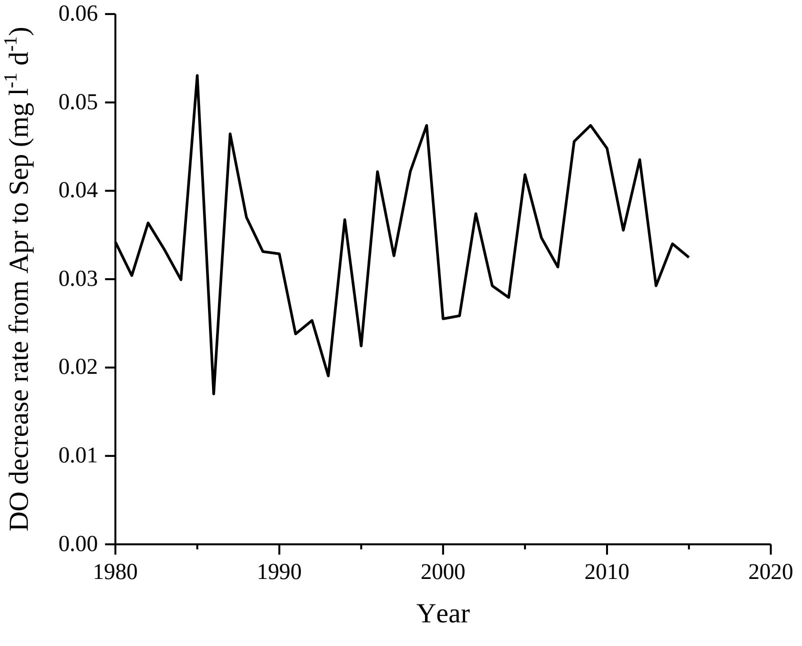

S-Fig. 5 Density difference between bottom water at the Jan 1st survey and water with the same temperature as the air in Mar vs. time of disturbance.

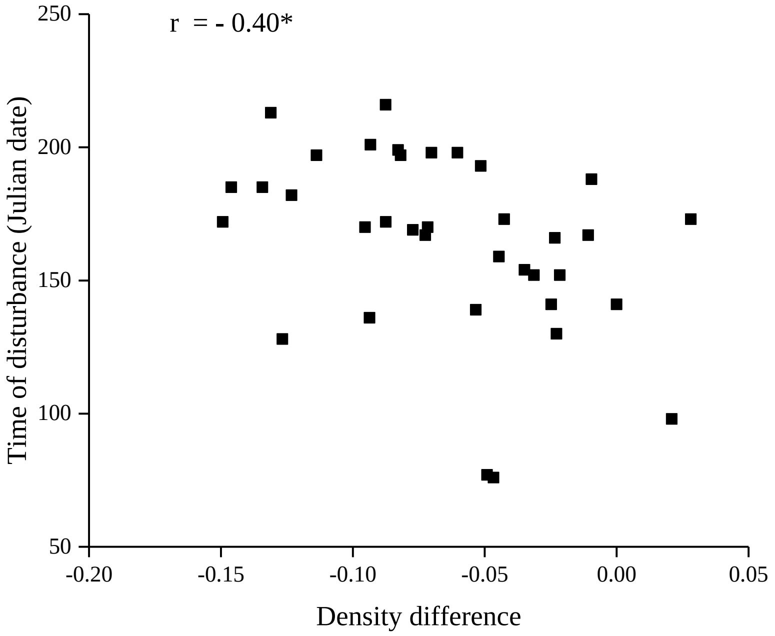

S-Fig. 6 Changes in water quality in the north basin of Lake Biwa. (1) BOD; (2) chlorophyll *a*; (3) TP. Red points indicate the averages determined by sequential t-test regime shift detection software (see text).

(1)

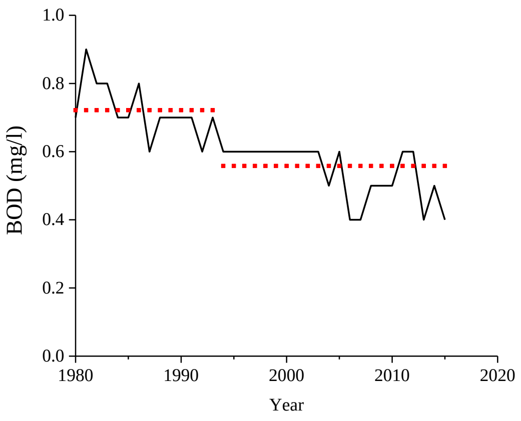

(2)

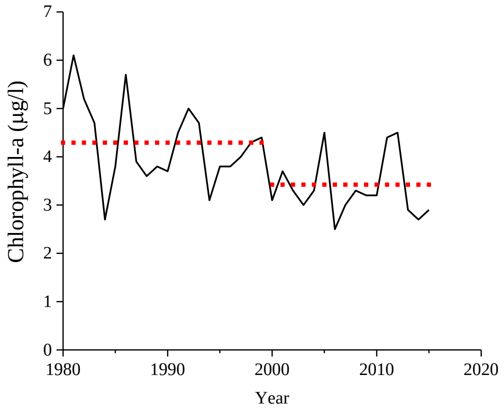

(3)

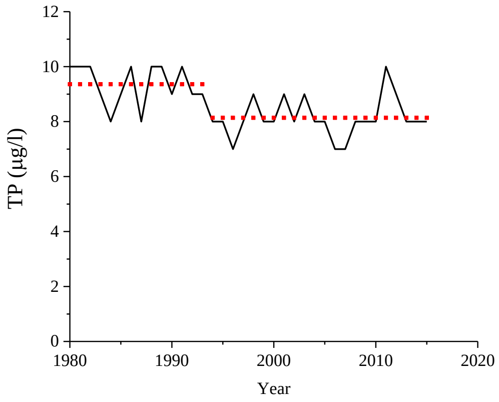

S-Fig. 7 Relation between DOmin at St. 1 and DOmin at St. 2.

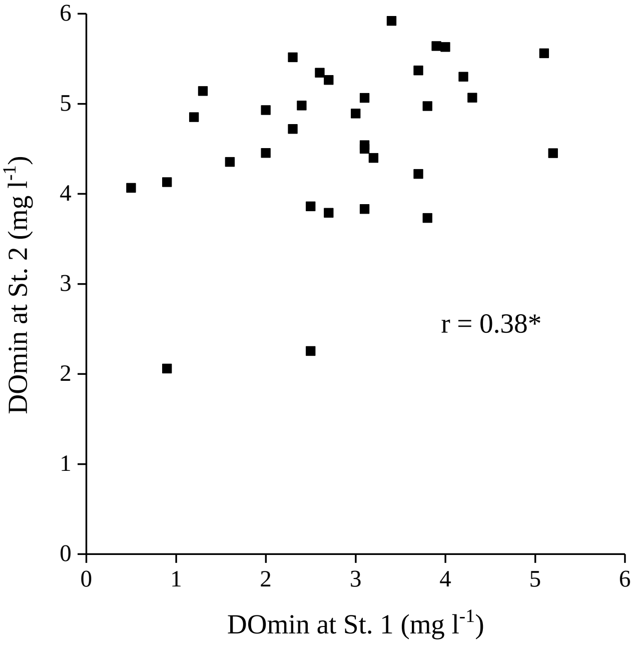

S-Fig. 8 Air temperature averaged from Sep to Dec in the preceding year vs. WT at bottom at the Jan 1st survey

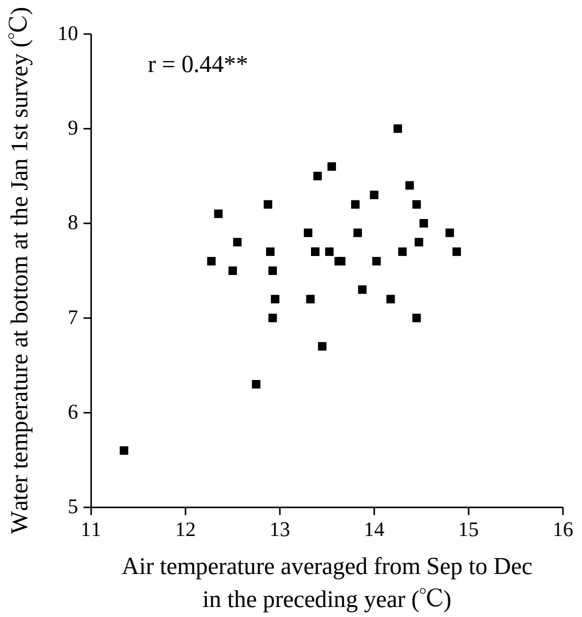

S-Fig. 9 Relation between DOmin observed and DOmin predicted using eqs. (3) & (4).

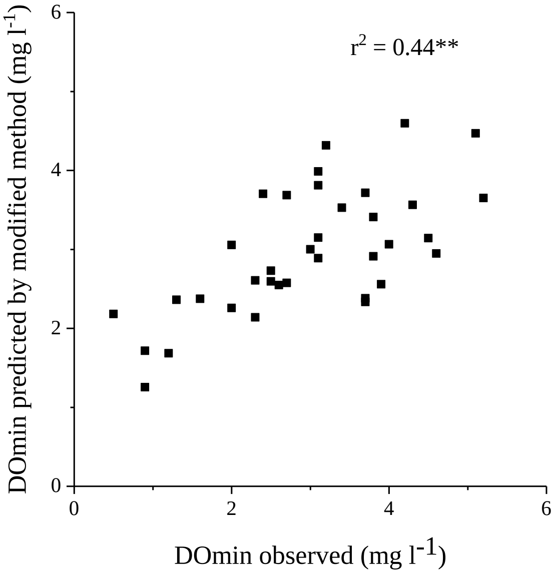

S-Table 1 Means and standard deviations of related water quality (measured by Shiga Prefectural Fisheries Experiment Station) and meteorological parameters in the three divided periods and significance of differences.

	A: 1963-1979	B: 1980-1993	C: 1994-2012	A vs B	B vs C	A vs C
DOmin (mg l^{-1})*1	5.49 ± 1.36	4.78 ± 0.93	4.50 ± 0.82			$P<0.05$
WTmin (℃)*2	6.74 ± 0.50	7.36 ± 0.97	7.72 ± 0.38	$P<0.05$		$P<0.01$
WTav (℃)*3	6.72 ± 0.47	7.23 ± 0.78	7.65 ± 0.34	$P<0.05$	$P<0.05$	$P<0.001$
NO_3-N (mg l^{-1})*3	0.132 ± 0.050	0.208 ± 0.045	0.252 ± 0.015	$P<0.001$	$P<0.001$	$P<0.001$

*1: yearly minimum DO at bottom, *2: WT at bottom at minimum DO observed, *3: annual average at bottom
